# Emotional self-states and coping responses in patients with chronic tinnitus: a schema mode model approach

**DOI:** 10.3389/fpsyt.2024.1257299

**Published:** 2024-02-21

**Authors:** Benjamin Boecking, Eva Stoettner, Petra Brueggemann, Birgit Mazurek

**Affiliations:** Tinnitus Center, Charité – Universitätsmedizin Berlin, Corporate Member of Freie Universität Berlin and Humboldt Universität zu Berlin, Berlin, Germany

**Keywords:** tinnitus, schema therapy, schema-mode-model, perceived stress, anxiety, depression, tinnitus-related distress

## Abstract

**Background:**

Amongst “third-wave” cognitive behavioural therapies, schema therapy demonstrates encouraging efficacy across various mental health conditions. Within this field, clinical interest has begun to converge on the “schema-mode-model” – a conceptualization framework for affective, cognitive and behavioral states that guide individuals’ perceptions and behaviours at a given point in time. Schema mode expressions in patients with chronic tinnitus are as-yet unexamined.

**Methods:**

The present study reports self-report data from *N* = 696 patients with chronic tinnitus who completed the Schema Mode- and Tinnitus Handicap Inventories alongside measures of perceived stress, anxiety and depression. The Schema Mode Inventory assesses so-called maladaptive “parent”, “child” and “coping” modes. Parent modes can be understood as self-states which are characterized by self-critical and hostile beliefs; child modes are characterized by biographically unmet emotional needs; and coping modes by inflexible attempts to regulate emotion and stabilize one’s sense of self. Descriptive, correlational and mediation analyses investigated schema mode expressions (1) in patients with chronic tinnitus, (2) as compared to published reference values from a healthy control sample, (3) in their relation to other psychological constructs, and (4) regarding their potential role in driving tinnitus-related distress.

**Results:**

Patients reported mild-to-moderate levels of emotional distress. Compared to healthy controls, patients showed (1) high relative expressions of child-, detachment and compliant coping modes and (2) a conspicuously low relative expression of the ‘punitive parent’ mode. Correlational patterns suggested strong associations of (1) parent as well as angry child modes with perceived stress and anxiety, (2) the vulnerable child mode with all measured constructs and (3) emotional distress with - intrapersonally - emotional detachment as well as - interpersonally - alleged compliance. Mediation analyses demonstrated that tinnitus-related distress was driven by significant interactions between child and coping modes.

**Conclusions:**

The study provides initial clinical evidence for the relevance and applicability of schema-mode based formulation and treatment planning in patients with chronic tinnitus.

## Introduction

Schema therapy is a flexible and transdiagnostic psychological treatment approach (1) which was initially developed for the formulation and treatment of pervasive emotional difficulties. In recent years, researchers and clinicians have been increasingly interested in its usefulness as a general psychotherapeutic formulation and treatment framework. As a “third-wave” cognitive behavioural therapy (CBT), schema therapy integrates cognitive-behavioural, emotion-focused and humanistic principles based on cognitive as well as psychodynamic theory. Unlike ‘traditional’ CBT, schema therapy adopts an explicit focus on patients’ emotions and pays particular attention to the therapeutic relationship as well as links between autobiographical experiences and dynamic patterns of thoughts, feelings, and behaviours in the here-and-now (2).

Schema therapy is built on two central concepts (1): (1) “early maladaptive schemas” - deeply rooted psychological “themes”, which are characterized by certain patterns of unmet childhood needs and early attachment experiences; and (2) “schema modes” - momentarily activated, dynamic state expressions of the more trait-like schema personality features.

The schema therapy framework subsumes schema modes under so-called “parent”, “child”, and “coping” domains. In addition, a “healthy adult” mode reflects individuals’ ability to integrate thoughts, feelings and behaviours in a context-appropriate, flexible, and adaptive manner (2, 3).

Schema modes can be thought of as cognitive-affective-behavioural “self-states” wherein, at a given point in time, individuals’ thoughts, feelings and behaviours are determined by “parent modes” (negative self-related beliefs related to internalized early relational objects), “child modes” (beliefs and primary emotions associated with unmeet childhood needs), or “coping modes” (secondary emotions and maladaptive coping behaviours; 4, 5).

“Parent modes” are learnt cognitive-affective systems, which lead people to experience themselves in a manner akin to how close attachment figures treated them. These systems were initially learned through implicit and model learning (6) and self-perpetuate via their interaction with maladaptive child and coping modes. In “punitive parent” mode, patients may feel intolerant, impatient and cruel towards themselves or others - or guilty and ashamed of their alleged shortcomings at the time (6). In “demanding parent” mode, individuals may emotionally experience high levels of pressure to fulfill rigid norms and values and feel obliged to act accordingly in order to protect their sense of self.

“Child modes” represent self-states that are characterized by unmet emotional needs. The “vulnerable child” mode encompasses ways of thinking and feeling that reflect unmet emotional core needs in childhood. Its quality may differ according to idiosyncratic experiences in peoples’ autobiographies. For example, having experienced unpredictable or frightening parental behaviours, the vulnerable child may take the form of high levels of anxiety (and anger) in close relationships; neglect and ignorance may lead to feelings of loneliness or isolation; and guilt-inducing or blaming behaviours may lead to pervasive feelings of shame or lack of self-worth. The “angry child” aims to protect the vulnerable child mode and presents as a tendency to show spontaneous angry reactions to unmet needs (6).

“Maladaptive coping modes” are learnt behavioural states that initially helped children to adapt to difficult, negligent or emotionally harmful environments. These attempts to ensure a sense of (emotional) safety map onto strategies akin to “fight” (overcompensating modes; e.g. “self-aggrandizer” or “bully-attack”), “flight” (avoidant modes; e.g. “detached protector” or “detached self-soother”) or “freeze” responses (e.g. “compliant surrenderer”; 1). Once established, coping modes can maintain emotional distress in adulthood by becoming too rigid and inflexible – thereby preventing corrective emotional learning (6).

**Figure 1:**
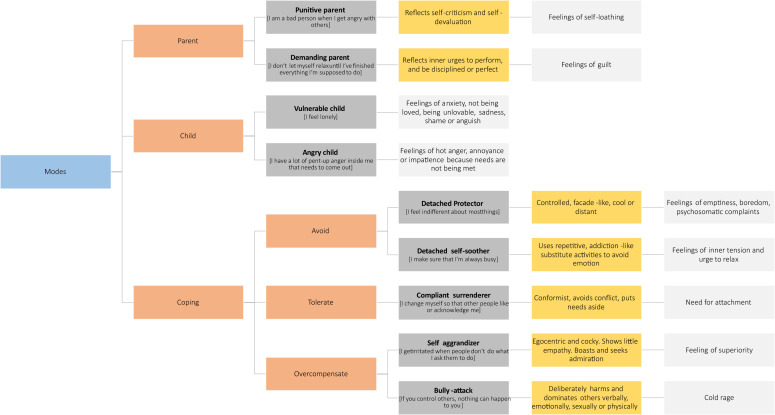
The schema-mode-model. Orange boxes indicate superordinate mode groups, dark grey boxes associated modes and exemplary measurement items, yellow boxes the “quality” of the mode as reflected in the actualized clinical patient presentation, and light grey boxes associated emotions or emotional needs respectively.

Whilst schema modes exist in everyone, clinical populations differ in terms of the frequencies and intensities with which maladaptive modes occur (1). Therapeutically, schema therapy adopts a variety of cognitive, behavioural, emotion-focused/experiential and relational treatment techniques contingent on the moment-by-moment occurrence of schema modes or mode-shifts, respectively. The therapeutic process aims to decrease the intensity and rigidity of maladaptive parent and coping modes, support the experience and expression of the child modes, and strengthen the healthy adult mode (7). **Figure 1** provides an illustration of the schema-mode-model as assessed in the present study.

Because both symptoms, broader psychological difficulties and problematic interpersonal patterns can be conceptualized as mode activations, mode interactions or mode 'flips', the schema-mode-model offers a highly flexible transdiagnostic framework for psychological formulation and treatment planning (2). Importantly, the model can be used both in a nomothetic and idiographic way – thereby offering a helpful tool to conceptualize psychological processes in a given patient population for clinical researchers – and in a single individual for psychological practitioners.

Schema mode expressions in patients with chronic tinnitus are unknown. Chronic tinnitus denotes a phantom perception of noise without an external sound source for a period of more than 3 months (8). Whilst acute tinnitus can be associated with a variety of factors, most importantly hearing loss (9), its chronic expression appears to denote an independent clinical entity – which is closely associated with psychological distress – primarily individual expressions of depression and anxiety (10–12). Research on personality predispositions that may increase an individual’s vulnerability to interpret the tinnitus symptom in a threatening manner is heterogeneous (13). This heterogeneity may be partly owed to a dissociation between individuals with or without identified inner-ear pathology one the one hand (where an underlying medical condition may explain initial symptom onset) – and idiosyncratic psychological vulnerability-stress profiles that influence the psychological ‘style’ in which the symptom is appraised on the other hand (14–17).

Initial research on the impact of coping styles in patients with chronic tinnitus also yielded mixed and somewhat contradictory results. For example, Budd and Pugh (18) identified that tinnitus-related distress was influenced by catastrophic thinking about the consequences of tinnitus – and associated avoidance behaviours. By contrast, embracing seemingly ‘adaptive’ coping styles (such as positive self-talk, distraction or attention switching techniques) did *not* predict lower levels of tinnitus-related distress. Postulating a warning against mechanistic ways of psychological thinking, the authors concluded that “*the mere use of coping strategies does not necessarily mean that these strategies will be useful*” (18, S. 334). This important conclusion was further corroborated by Beukes et al. (19), Dineen et al. (20), and Andersson and Willebrandt (21) all of whom emphasized that coping strategies “per se” do not effectively reduce tinnitus-related distress. Indeed, the mechanistic idea of “coping” in a standardisable matter without considering both context and individual psychological profiles appears rather unhelpful. Rather, research ought to focus on the role of behaviours’ *context*, *meaning* and *function* for individuals in order to understand psychological appraisal-emotion-reaction patterns.

## Method

### Participants

The present study reports self-report data from *N* = 696 patients who were sampled over a period of two years in routine clinical practice. Patients (a) self-referred to the Tinnitus Center at Charité Universitätsmedizin Berlin between January 2019 and December 2020, (b) suffered from chronic tinnitus (lasting for > 3 months), (c) were 18 years of age or older and (d) completed German versions of the revised Schema Mode Inventory (SMI-r), Tinnitus Handicap Inventory, Perceived Stress Questionnaire, and the Hospital Anxiety and Depression Scale. Exclusion criteria comprised the presence of acute psychotic illness or addiction, (untreated) deafness and insufficient knowledge of the German language. The sample featured equal gender proportions (51% female). Participants were between 19 and 82 years old (*M_age_* = 51.87 years; *SD* = 12.18). Upon arrival at the Tinnitus Center, patients completed a routine questionnaire assessment battery on electronic tablet devices. Participants provided written consent for data to be collected and used for research purposes, and the Charité Universitätsmedizin Berlin’s ethics committee approved data collection and analysis (No: EA4/216/20). **Table 1** provides an overview of the sample’s sociodemographic characteristics.

**Table 1:** Sociodemographic information (*N* = 696 patients with chronic tinnitus).

Variable	*M*	*SD*
Age	51.87	12.18
	***n***	***%***
Gender		
Male	338	48.6
Female	358	51.4
Duration of tinnitus		
*<*1/2 year	85	12.2
1/2–1 year	144	20. 7
1–2 years	97	13.9
2–3 years	46	6.6
3-4 years	38	5.5
*>*4 years	286	41.4
Education		
None	7	1.0
Primary school	9	1.3
General school	42	6.0
O-Levels	118	17.0
A-Levels	60	8.6
Apprenticeship	150	21.6
Polytechnic degree	76	10.9
University degree	234	33.6
Nationality		
German	640	92.0
Other	56	8.0
Relationship status		
Single	164	23.6
In Partnership	70	10.1
Married	341	49.0
Separated	15	2.2
Divorced	89	12.81
Widowed	17	2.4
Work status		
Employed	493	70.8
Unemployed	203	29.2

M, mean; SD, standard deviation.

### Measures

#### Schema Mode Inventory – revised version

The revised version of the Schema Mode Inventory (SMI-r) is a 124-item self-report questionnaire which measures emotions, cognitions and behaviours across a total of 14 schema modes (22). Because schema modes denote dynamically occurring self-states, the SMI-r assesses the frequency with which these occur. For the present study, we selected the 10 most common modes (1). Participants thus rated 86 items on a 6-point Likert scale from 1 = “*never or hardly ever*” to 6 = “*always*”. Scale scores were averaged into mode scores between 1 and 6 – with higher scores indicating higher frequency of mode manifestation. The German version of the SMI-r yields good-to-excellent internal consistency and construct validity (23). In the current sample, internal consistencies were acceptable-to-excellent (α_punitive parent_ = 0.84; α_demanding parent_ = 0.84; α_vulnerable child_ = 0.93; α_angry child_ = 0.84; α_detached protector_ = 0.86; α_detached self-soother_ = 0.72; α_compliant surrenderer_ = 0.73; α_self-aggrandizer_ = 0.79; α_bully and attack_ = 0.79; α_healthy adult_ = 0.81). The presently measured modes were^1^:

1. [maladaptive parent modes] the punitive parent (PP; restricts, criticizes, or punishes the self or others); and demanding parent modes (DPT; sets high expectations and high levels of responsibility towards self and others; pressures self or others to achieve expectations);
2. [maladaptive child modes] the vulnerable (VC; experiences unhappy or anxious emotions, especially fear, sadness and helplessness) and angry child modes (AC; feels intensely angry, infuriated, frustrated or impatient, because the emotional needs of the vulnerable child are not being met. Vents anger in inappropriate ways. May make demands that seem entitled or spoiled and that alienate others);
3. [maladaptive ‘avoidant’ coping modes] the detached protector (DP; withdraws or disconnects emotionally or demonstrates behavioural avoidance) and detached self-soother modes (DSS; uses repetitive, ‘addictive’, compulsive or pleasurable behaviours to calm, soothe, and distance self from painful feelings);
4. [maladaptive ‘surrender’ coping mode] the compliant surrenderer mode (CS; seemingly complies with others’ expectations; adopts a subordinate, passive, dependent or passive-aggressive stance);
5. [maladaptive ‘overcompensation’ coping modes] the self-aggrandizer (SA; feels superior, special, or powerful; looks down on others; shows off or acts in a self-important, self-aggrandizing manner; concerned about appearances rather than feelings or real contact with others) and bully-and-attack modes (BA; threatens, coerces or intimidates others to get what they want, may retaliate against others, or assert their dominant position; feels a sense of sadistic pleasure in attacking others); and
6. [adaptive mode]: the healthy adult mode (HA; nurtures, affirms and protects the VC mode, sets limits for the AC mode and moderates the dysfunctional coping and parent modes).

#### Tinnitus Handicap Inventory (THI)

Tinnitus-related distress, i.e. *subjective tinnitus handicap* severity was measured by the German version (24) of the Tinnitus Handicap Inventory (27). The scale consists of 25 items that are answered on a 3-point scale (0 = *no*; 2 = *sometimes*; 4 = *yes*) resulting in a total score between 0 and 100. In the current sample, the measure’s internal consistency was excellent (α = 0.93).

#### Perceived Stress Questionnaire

The Perceived Stress Questionnaire (28, 29) assesses subjective stress experiences across four dimensions three of which measure people’s internal stress reaction (tension [disquietude, exhaustion and lack of relaxation], worries [anxious concern for the future, and feelings of desperation and frustration], and joy [positive feelings of challenge, joy, energy, and security]). The fourth scale measures perceived external stressors (demands [perceived environmental demands such as lack of time, pressure, and overload]). The scale consists of 30 items that are rated on a 4-point scale (1 = *almost never*, 2 = *sometimes*, 3 = *often*, 4 = *almost always*). All indices are linearly transformed to range from 0 to 100. All scores are averaged into a total score for which *joy* is recoded. In the current sample, internal consistency was excellent (α = 0.94).

#### Hospital Anxiety and Depression Scale–German version

Anxiety and depressive symptoms were measured using the Hospital Anxiety and Depression Scale (30, 31). The questionnaire features two 7-item scales that assess anxious or depressive symptoms “during the last week” (0 = “*not at all*” to 3 = “*mostly*”). For the current sample, internal consistencies were good (α_anxiety_ = 0.80; α_depression_ = 0.88).

### Data analysis

**Figure 2:**
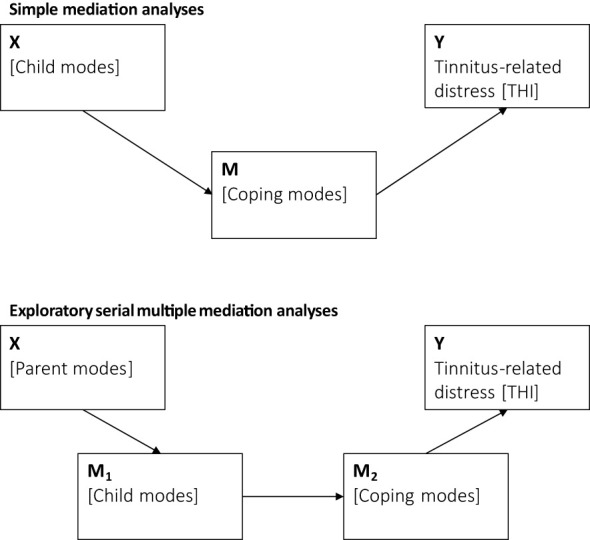
Simple and serial mediation models that examine the role of schema modes and tinnitus-related distress.

Analyses were conducted using IBM SPSS Statistics for Windows, Version 24. First, we report descriptive statistics and correlation coefficients *r*. Second, we compared SMI-r scores with normative reference data from a healthy German convenience sample (23, p. 301). Effect sizes Cohen’s *d* were calculated (32) with estimates being defined as small (0.20–0.49), medium (0.50–0.79) or large [> 0.80; (33)]. Third, mediation analyses (34) explored the role of schema modes for the quality or maintenance of tinnitus-related distress. Following theoretical considerations wherein (1) child modes drive coping modes, or (2) maladaptive parent modes drive child modes – which are then coped with (1), two sets of mediation analyses were conducted:

First, simple mediation models specified child modes as independent (X), coping modes as mediating (M), and tinnitus-related distress as dependent variables (Y). Second, exploratory analyses examined serial multiple mediation models wherein parent modes were specified as independent (X), child modes as first-step mediating (M_1_), coping modes as second-step mediating (M_2_), and tinnitus-related distress as dependent variables (Y). Due to the exploratory nature of the analyses in our patient population, significance levels were set to 0.05. See **Figure 2** for an illustration of the examined indirect-effects models.

## Results

### Descriptive indices

**Table 2** reports means and standard deviations for the completed questionnaire measures as well as mean differences between the current sample’s SMI-r scale scores and published reference values for healthy participants from a German reference sample (23, **Table 4**). Overall, patients reported moderate-to-severe levels of tinnitus-related distress and mild-to-moderate levels of perceived stress, anxiety, and depressivity.

**Table 2:** Means and standard deviations for the examined questionnaires for *N* = 696 patients with chronic tinnitus.

	*M*	*SD*			
THI	45.35	22.17			
PSQ	50.97	20.19			
worries	43.04	24.15			
tension	59.29	23.53			
joy	46.51	23.61			
demands	48.07	24.70			
HADS_a	8.53	4.14			
HADS_d	7.07	4.68			
SMI-r			M_reference_ (23)	SD_reference_ (23)	***d***
PP	1.71	0.63	2.59	0.98	-1.18
DPT	3.00	1.03	2.59	0.98	0.41
VC	2.16	0.95	1.49	0.69	0.75
AC	2.27	0.79	1.79	0.66	0.63
DP	2.15	0.77	1.40	0.62	0.60
DSS	2.77	0.99	2.16	1.04	0.61
CS	2.77	0.73	2.34	0.80	0.57
SA	2.29	0.67	2.17	0.66	0.18
BA	1.65	0.57	1.46	0.49	0.34
HA	4.26	0.75	4.65	0.63	-0.54

M, mean; SD, standard deviation; d, Cohen’s d (|d| ≤ 0.2, negligible effect; 0.2 > |d| ≤ 0.5, small effect; 0.5 > |d| ≤ 0.8, medium effect; and |d| > 0.8, large effect); PP, punitive parent mode; DBT, demanding parent mode; VC, vulnerable child mode; AC, angry child mode; DP, detached protector mode; DSS, detached self-soother mode; CS, compliant surrenderer mode; SA, self-aggrandizer mode; BA, bully-attack mode; HA, healthy adult mode; HADS_a, Hospital Anxiety and Depression Scale-Anxiety subscale; HADS_d, Depression subscale; PSQ, Perceived Stress Questionnaire (German version); THI, Tinnitus Handicap Inventory (German version); SMI-r: Schema Mode Inventory-revised (German version). The lower part of the table reports between-group comparisons with published reference values of *N* = 432 healthy controls (23, **Table 4**).

**Table 3** reports Pearson’s correlation coefficients (*r*) - in particular for the schema modes and the other obtained measures (Panel 3). Results suggest that (1) parent and angry child modes drive perceived stress and anxiety, (2) the vulnerable child mode underlies most measured constructs and (3) patients appear to cope with emotional distress by means of emotional detachment (intrapersonally) or ‘compliant surrendering’ (interpersonally).

**Table 3:** Significant Pearson correlation coefficients for the commonly measured constructs (Panel 1), the SMI-r (Panel 2), and the SMI-r with the other measured constructs (Panel 3).

Panel 1:							
	PSQ					HADS_a	HADS_d
	total	worries	tension	joy	demands		
THI	0.59	0.59	0.55	-0.53	0.31	0.63	0.66
total		0.88	0.89	-0.84	0.76	0.73	0.70
worries			0.71	-0.71	0.54	0.73	0.69
tension				-0.71	0.59	0.70	0.61
joy					-0.42	-0.63	-0.77
demands						0.41	0.29
HADS_a							0.69

Panel 2:									
SMI-r	DPT	VC	AC	DP	DSS	CS	SA	BA	HA
PP	0.58	0.72	0.59	0.63	0.47	0.51	0.44	0.49	-0.46
DPT		0.52	0.51	0.45	0.50	0.50	0.56	0.35	-0.10
VC			0.66	0.73	0.45	0.54	0.35	0.38	-0.50
AC				0.53	0.49	0.41	0.55	0.58	-0.28
DP					0.41	0.48	0.33	0.41	-0.42
DSS						0.41	0.40	0.37	-0.14
CS							0.27	0.23	-0.28
SA								0.60	–
BA									-0.18

Panel 3:								
SMI-r	THI	PSQ					HADS_a	HADS_d
		total	worries	tension	joy	demands		
PP	0.42	0.53	0.57	0.44	-0.46	0.30	0.53	0.47
DPT	0.34	0.60	0.56	0.53	-0.40	0.54	0.52	0.36
VC	0.52	0.67	0.72	0.56	-0.66	0.32	0.64	0.68
AC	0.40	0.53	0.58	0.44	-0.44	0.33	0.50	0.43
DP	0.43	0.54	0.54	0.47	-0.58	0.24	0.50	0.61
DSS	0.36	0.40	0.43	0.39	-0.27	0.27	0.45	0.32
CS	0.30	0.41	0.43	0.37	-0.35	0.24	0.36	0.36
SA	0.19^*^	0.31	0.32	0.25	-0.16	0.29	0.28	0.14
BA	0.22	0.24	0.31	0.17	-0.18	0.15	0.26	0.23
HA	-0.40	-0.39	-0.40	-0.32	0.47	-0.13	-0.40	-0.47

* = p <.05; Light grey cells indicate small (|r| ≤ 0.29 = small correlation), yellow medium (0.30 > |r| ≤ 0.49), and orange large correlations (0.50 > |r| ≤ 1.0). PP, punitive parent mode; DBT, demanding parent mode; VC, vulnerable child mode; AC, angry child mode; DP, detached protector mode; DSS, detached self-soother mode; CS, compliant surrenderer mode; SA, self-aggrandizer mode; BA, bully-attack mode; HA, healthy adult mode; THI, Tinnitus Handicap Inventory; PSQ, Perceived Stress Questionnaire; HADS_a, Hospital Anxiety and Depression Scale-Anxiety subscale; HADS_d, Depression subscale; SMI-r: Schema Mode Inventory-revised. Unless otherwise indicated, all coefficients are *p* <.001.

### Comparisons of SMI-r data with normative data from a healthy German reference sample

**Figure 3:**
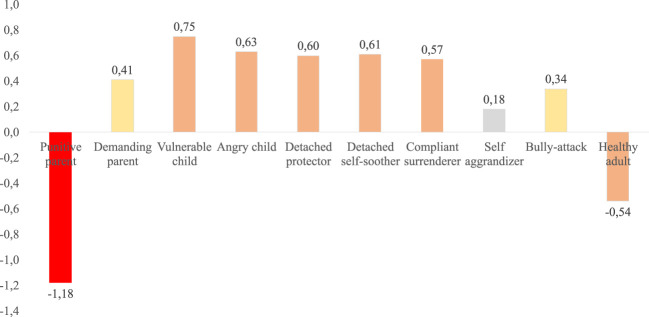
Relative schema mode expressions in the current patient sample compared to published reference values from a healthy-control reference sample. The Y-Axis illustrates effect sizes *d*. Negative values denote schema expressions that are lower than those reported in the reference sample. Positive values denote schema expressions that are higher than those reported in the reference sample. Light grey bars indicate negligible (|d| ≤ 0.19), yellow small (0.20 > |d| ≤ 0.49), orange medium (0.50 > |d| ≤ 0.79), and red large effect sizes (|d| > 0.80).

**Figure 3** illustrates the relative expressions of schema mode expressions in the current sample of *N* = 696 patients with chronic tinnitus compared to published reference values from *N* = 432 healthy controls (23, **Table 4**). Overall, patients with chronic tinnitus show (1) high relative expressions of child as well as avoidant and surrendering coping modes and [2] a conspicuously low relative expression of the punitive parent mode (cf. also **Table 2**).

**Table 4:** Significant indirect effect coefficients from the simple (upper part) and exploratory serial multiple mediation models (lower part).

X:	M:		Y:				
Child mode	Coping mode		Tinnitus-related distress [THI]	Effect	Boot SE	Boot LLCI	Boot ULCI
VC	DP			0.069	0.035	0.002	0.138
	DSS			0.068	0.018	0.034	0.104
AC	DP			0.159	0.023	0.115	0.206
	DSS			0.104	0.020	0.067	0.147
	CS			0.066	0.017	0.034	0.101
X:	M_1_:	M_2_:	Y:				
Parent mode	Child mode	Coping mode	Tinnitus-related distress [THI]				
PP	VC	DSS		0.023	0.008	0.009	0.041
	AC	DP		0.032	0.009	0.016	0.052
		DSS		0.029	0.009	0.014	0.047
		BA		-0.022	0.011	-0.043	-0.001
DPT	VC	DSS		0.019	0.006	0.008	0.031
	AC	DP		0.057	0.011	0.037	0.080
		DSS		0.028	0.008	0.014	0.044
		CS		0.012	0.005	0.003	0.023

Coefficients are completely standardized. Boot, Bootstrap (10000 iterations); SE, standard error; LLCI, lower level confidence interval; ULCI, upper level confidence interval; PP, punitive parent mode; DBT, demanding parent mode; VC, vulnerable child mode; AC, angry child mode; DP, detached protector mode; DSS, detached self-soother mode; CS, compliant surrenderer mode; BA, bully-attack mode; THI, Tinnitus Handicap Inventory.

### Mediation analyses

Next, we followed up on the modes that showed at least a "small" difference in effect size compared to the general population. We specified two sets of mediation analyses wherein we computed

- two [X: vulnerable or angry child] x 4 [M: detached protector, detached self-soother, compliant surrenderer, bully-attack] and, exploratorily,
- two [X: punitive or demanding parent] x 2 [M_1_: vulnerable or angry child] x 4 [M_2_: detached protector, detached self-soother, compliant surrenderer, bully-attack] models. In all models, the dependent variable was tinnitus-related distress as measured by the THI.

Results suggested that tinnitus-related distress was driven by an interaction of – strong – feelings of vulnerability and anger associated with unmet emotional needs – that tended to be coped with through emotional detachment or (seeming) subordination. Exploratory analyses further suggested that the parent modes may influence such processes - although the reader is reminded that the punitive parent mode is conspicuously *under*expressed in the present sample. **Table 4** lists the indirect effect coefficients.

## Discussion

This study is the first to examine schema modes, i.e. autobiographically shaped and situationally activated “self-states” (35) in patients with chronic tinnitus. Schema modes are part of a transdiagnostic psychological formulation and treatment framework that has been shown to be successful in ameliorating emotional distress across a broad variety of psychological difficulties (26, 36–38).

First, we compared the frequencies and intensities of schema mode activations of patients with chronic tinnitus to those of healthy controls from a German-speaking reference sample (23). We found that patients with chronic tinnitus showed frequent activation of the vulnerable and angry child modes, indicating high levels of latent emotional vulnerability and painful emotions, such as fear, sadness, shame, loss and anger in our sample.

To cope with these emotions, patients showed frequent activation of avoidant/detached coping modes, which involve affective numbing and avoidance of emotional themes, feelings, or thoughts. Consistent with clinical observation, chronic tinnitus patients in avoidant coping modes might either be unaware of their emotions - or reluctant to engage with them therapeutically. In clinical contact, patients might present with various subtle forms of emotional detachment, such as (1) intellectualization, (2) distant, superficial, or overly ‘cognitive’ reflections, (3) vagueness, (4) avoidance of meaningful or emotional topics and (5) a demand for concrete “solutions” rather than allowing for a focus on emotional topics and themes.

Interpersonally, the compliant surrenderer dominated patients' emotional coping styles. Correspondingly, patients did not commonly endorse the more overtly narcissistic self-aggrandizer. The compliant surrenderer mode reflects a tendency to please and conform to others’ expectations at the expense of one’s own needs - but also to experience or demonstrate heightened levels of anxiety, confusion or passive-aggression if others do not meet one’s expectations. In psychological therapy, patients in this mode may seem passive and submissive, but also undermine the therapist or the therapeutic process in subtle ways in order to indirectly express anger or assert a sense of control.

A less common but still frequently expressed coping mode was the bully-attack, which presents as patients demonstrating an aggressive demeanor with a goal to intimidate others in order to protect themselves from perceived threat. In this mode, patients may threaten third-parties including clinicians directly or indirectly in order to assert power or dominance. The mode can range from subtly menacing or threatening implications to overt attacks at the extreme end of the spectrum.

Surprisingly, patients with chronic tinnitus hardly ever endorsed statements linked to the punitive parent mode. Whilst this conspicuously low frequency of the punitive parent may reflect a “true” absence of respective beliefs, the finding is much more likely to reflect patients’ detached coping style indicating an emotional avoidance of key themes pertaining to vulnerability, anger, shame or stigma (39). This interpretation appears likely given that (1) the punitive parent and detached protector mode correlated highly, and (2) depression [which drives tinnitus chronification (11)] is also characterised by frequent expresisons of punitive parent, child, and avoidant coping modes (40, 41).

Correlational analyses revealed moderate-to-strong correlations between the vulnerable child and all psychological distress-related constructs; as well as the angry child and perceived stress, anxiety and worry. Beyond the helpful therapeutic focus this may offer, these findings are in keeping with previous research on “worry as avoidance” of underlying, harder-to-bear emotional states (42, 43).

Intrapersonally, patients with chronic tinnitus seem to cope primarily with emotional detachment – in particular the detached protector mode. Whilst emotional detachment may be useful in the short term, its highly negative correlation with ‘joy’ also emphasizes the emotional “price to pay” for this self-protection strategy, i.e. that feelings of joy are numbed alongside emotions that are perceived as more threatening (44). Because the detached protector *involves* ‘cognitive’ ways of dealing with emotional distress (e.g. being overly rational, intellectualizing or adopting a ‘somatic’ focus), cognitive interventions may maintain tinnitus-related distress. By contrast, *emotion*-focused techniques may offer a more helpful group of psychological treatment techniques to ameliorate distress (45). Overall, these findings are in keeping with evidence on associations between medically unexplained symptoms and difficulties in affect regulation (46) and suggest that an affective focus in psychological therapy may be well suited to ameliorate tinnitus-related distress (47).

Interpersonally, patients appeared to manage emotion-driven self-states most frequently in the compliant surrenderer mode. In this mode, patients may either act in seemingly passive, approval-seeking or self-deprecating ways or disempower themselves by showing inflexible self-pitying tendencies. This interaction style may lead to complex interpersonal dynamics in the multiprofessional team as well as patients’ lives that should be thoroughly assessed and addressed throughout psychological therapy (48, 49).

Overall, the results mirror previous findings on “emotional excitability” (potentially reflecting child modes) on the one hand and “aggression inhibition” on the other hand (potentially reflecting detached or avoidant coping modes) as personality-rooted drivers of tinnitus-related distress (50).

Cross-sectional mediation analyses suggested that tinnitus-related distress was driven by interactions of child and avoidant coping modes. Clinically, this finding suggests that psychological interventions should focus less on avoidance of “the tinnitus symptom” or “behaviours that are avoided because of tinnitus”, but on underlying *emotional themes* pertaining to feelings of vulnerability, shame or anger. The idea of emotional avoidance as a key transdiagnostic mediator of stress experiences and psychological difficulties has been well researched (51, 52); however, the present study is the first to demonstrate a comparable construct from the schema-mode-model in patients with chronic tinnitus.

The compliant surrender’s role in maintaining tinnitus-related distress reflects existing research on challenging interpersonal dynamics in patients presenting with functional syndromes or depression respectively (53, 54). The mode’s mediating role further suggests that a relational focus on patients’ emotion regulation strategies may further benefit psychological treatment outcome. This is in keeping with previous research that suggests an interplay of emotional detachment and seemingly pleasing (yet controlling) interpersonal coping styles in patients with functional somatic syndromes (55) - and a therapeutic need to adopt both intrapersonal and interpersonal perspectives regarding patients’ emotions and biographically influenced, dynamically applied regulation strategies.

Exploratory models that included the punitive and demanding parent modes resulted in broadly similar patterns emphasizing the crucial importance of underlying feelings of vulnerability and anger – and emotional detachment or interpersonal compliance as attempts to stabilize the self at high cost. Notably, one indirect effect suggested positive associations between the punitive parent, angry child, and bully-attack mode – and a *negative* association between the latter and tinnitus-related distress. For a subset of patients, it thus appears as if a coping style akin to “attacking others before they attack me” may protect them from feelings of vulnerability and increase senses of self-efficacy and control in the short-term (56).

## Limitations

The study features several methodological shortcomings. The absence of a “real” control group leaves the presented findings tentative: Whilst the healthy control reference sample was adequately powered, it was not representative of the general population (23) – and the interpretation of the here-reported between-group comparisons needs to be made with caution. Due to the cross-sectional nature of the data, the mediation analyses do not reflect “true” mediation – which requires temporal lags between the independent, mediating and outcome variables (57). Results can thus not be interpreted causally, and future prospective studies need to demonstrate the diagnostic usefulness and clinical relevance of the schema-mode-model for helping patients with chronic tinnitus-related distress.

In conclusion, schema therapy and its formulation and treatment framework offer a promising ‘modern’ clinical development in psychotherapy research and practice (58) with an ever-evolving empirical evidence base (5, 59). As a “third-wave” cognitive-behavioural therapy, it offers a broad, non-stigmatizing and holistic perspective on how to understand and ameliorate emotional suffering. Furthermore, its flexible conceptualization framework enables practitioners to use a wide range of emotion-focused, and humanistically informed experiential interventions in clinical practice. The present study is the first to demonstrate (1) the expression and interaction of maladaptive schema modes in patients with chronic tinnitus and (2) strong correlations between these self-states and more established psychological constructs. The findings (1) suggest that tinnitus-related distress can be expressed in schema mode terms – which may offer a helpful framework for clinical formulation and treatment planning, and (2) open up schema-therapy based psychotherapeutic interventions for empirical examination in in this population (26).

## Data availability statement

As per Charité Universitätsmedizin Berlin’s ethics committee, unfortunately, we cannot make the data public without restrictions because we did not obtain patients’ consent to do so at the time. Nevertheless, interested researchers can contact the directorate of the Tinnitus Center with data access requests (birgit.mazurek@charite.de). Alternatively, interested researchers may also contact the Charité's Open Data and Research Data Management Officer Dr. Evgeny Bobrov (evgeny.bobrov@charite.de).

## Ethics statement

The studies involving humans were approved by Charité Universitätsmedizin Berlin EA4/216/20. The studies were conducted in accordance with the local legislation and institutional requirements. The participants provided their written informed consent to participate in this study.

## Author contributions

BB: Conceptualization, Data curation, Formal analysis, Methodology, Project administration, Visualization, Writing – original draft, Writing – review & editing. ES: Formal analysis, Writing – review & editing. PB: Writing – review & editing. BM: Funding acquisition, Resources, Writing – review & editing.

## Footnotes

1 Mode descriptions are paraphrased from (1, 5, 25, 26).
